# Uncovering the hidden risk of metastatic cutaneous basal cell carcinoma by molecular profiling: A retrospective review

**DOI:** 10.1016/j.jdin.2024.05.004

**Published:** 2024-05-24

**Authors:** Amanda J. Nguyen, Zachary C. Fogarty, Jaime Davila, Svetomir N. Markovic, Chen Wang, Ruifeng Guo

**Affiliations:** a Department of Laboratory Medicine and Pathology, Mayo Clinic, Rochester, Minnesota; b Department of Quantitative Health Sciences, Mayo Clinic, Rochester, Minnesota; c Department of Mathematics, Statistics, and Computer Science, St. Olaf College, Northfield, Minnesota; d Department of Oncology, Mayo Clinic, Rochester, Minnesota; e Department of Laboratory Medicine and Pathology, Mayo Clinic, Jacksonville, Florida

**Keywords:** Hedgehog inhibitors, metastatic basal cell carcinoma, molecular profiling, mutational analysis, *PTCH1*, UV mutation signature

*To the Editor:* Metastatic cutaneous basal cell carcinoma (BCC) is a rarely reported phenomenon, estimated to account for less than 0.01% of cases.^1^ However, the high prevalence of primary BCC makes it essential to consider the possibility of metastasis, which may be potentially underdiagnosed due to biased clinical assumptions that BCC is localized and indolent. While the distinction between BCC and squamous cell carcinoma (SCC) is often straightforward, they can occasionally show overlapping histopathologic features (Fig 1). Accurate diagnosis and differentiation from mimicking conditions is imperative, as advanced-stage patients can greatly benefit from Hedgehog pathway inhibitors (HhI) and immune checkpoint inhibitors (ICIs).^2^

**Fig 1:**
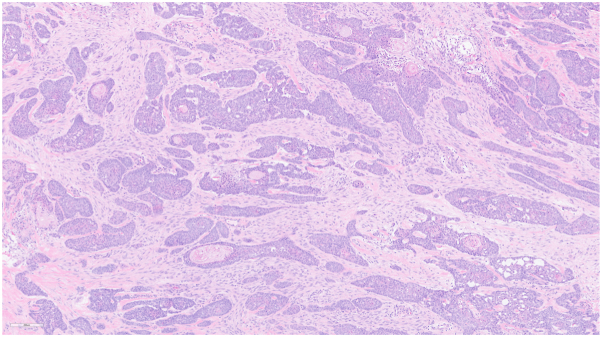
Infiltrative basal cell carcinoma (hematoxylin-eosin stain, 10×) can show overlapping histopathologic features with squamous cell carcinoma.

In a review of 123 cases initially diagnosed as metastatic SCC (Table I), subsequent large cancer mutation profiling identified 5 cases exhibiting high tumor mutation burden (TMB), ultraviolet (UV) type mutation signatures, and *PTCH1* gene mutations—molecular patterns consistent with BCC. This prompted a reevaluation of the patients’ medical records and pathology slides, resulting in confirmation of all 5 cases as metastatic BCC. After identifying these initially misdiagnosed cases, we expanded our review to identify additional cases, yielding an additional 5 metastatic BCCs and 2 aggressive primary BCCs. Among these cases, definitive BCC diagnosis was achieved only in the 2 primary tumors; 3 cases had metastatic BCC in the differential diagnosis, and the other 2 cases were favored to be basosquamous carcinoma and oropharyngeal SCC.

**Table I:** Summary of clinical features, mutational analysis, and clinical courses

Case	Sex	Age at diagnosis	Metastatic site(s)	Primary site, if known	TMB (Muts/Mb)	Mutation signature	Recurrent genes	Treatments	Follow-up
Metastatic basal cell carcinoma									
*1*	M	72	Parotid gland, lymph nodes, lung	Left postauricular skin	High (48)	UV	*PTCH1*	HHI, ICI	CR
2	M	65	Lung	Right cheek skin	High (32)	UV	*MLL2, TERT* promoter	HHI, ICI, TKI	DOD
*3*	M	61	Lung	-	High (40)	UV	*PTCH1, TERT* promoter*, TP53*	ICI	CR
*4*	M	53	Lung	-	High (42)	UV	*PTCH1, TERT* promoter	ICI	CR
*5*	M	67	Lymph node	Left arm skin	High (67)	UV	*MLL2, PTCH1, TERT* promoter*, TP53*	HHI, ICI	CR
6	M	80	Lung, lymph node	Left preauricular skin	High (106)	UV	*TERT* promoter*, TP53*	ICI	PR
7	M	36	Bone, lung	Right cheek skin	Intermediate (8)	UV	*PTCH1, TERT* promoter	EGFR inhibitor, HHI, ICI	SD
8	M	48	Bone, lung	Left shoulder skin	NP	NP	*MLL2, TERT* promoter	HHI, ICI	CR
*9*	M	68	Neck skin	-	High (81)	UV	*MLL2, PTCH1*	-	CR
10	F	73	Lymph nodes	Upper back skin	High (32)	UV	*MLL2, PTCH1, TERT* promoter*, TP53*	ICI	PR
Locally aggressive basal cell carcinoma									
11	M	74	-	Back skin	High (13)	UV	*PTCH1, TERT* promoter	HHI, ICI	PR
12	M	40	-	Eyelid skin	High (105)	UV	*PTCH1, TERT* promoter*, TP53*	HHI, ICI	CR
HPV-associated basaloid squamous cell carcinoma									
13	M	66	Lung, bone	Tonsil	Low (1)	NP	*NOTCH1*	EGFR inhibitor, HHI, ICI	DOD
14	F	58	Lung, diaphragm	Anal canal	Low (4)	NP	*NOTCH1*	ICI	PR
Non-skin squamous cell carcinoma									
15	M	56	Lymph node	Lung	High (25)	Tobacco	*CDKN2A/B, PIK3CA, TP53*	ICI	DOD
16	F	64	-	Lung	High (30)	APOBEC	*PIK3CA, RB1, TP53*	-	DOD
17	M	67	Supraclavicular region, brain, bone	Lung	High (25)	APOBEC	*CDKN2A, PIK3CA, TP53*	ICI	PR
18	F	61	Scalp skin, lymph nodes	Lung	High (24)	APOBEC	*CDKN2A/B, NOTCH1, PIK3CA, TP53*	ICI	PR
19	F	62	Pleura, lymph nodes	Lung	High (37)	Tobacco	*ARID1A, BRAF, KEAP1, MYC, PREX2, STK11, TP53, TSC2*	-	DOD
20	F	56	Groin region, brain	Cervix	High (26)	APOBEC	-	-	DOD
Cutaneous squamous cell carcinoma									
21	M	49	Mastoid, lymph node, brain		High (146)	UV	*RB1, TERT* promoter*,TP53*	EGFR inhibitor, ICI	DOD
22	M	62	Lymph nodes		High (76)	UV	*MLL2, NOTCH1, RB1, TP53*	ICI	DOD
23	M	77	Orbit, lung	Temple skin	High (59)	UV	*CDKN2A, TERT* promoter*, TP53*	ICI	PR
24	M	79	Lymph nodes		High (87)	UV	*CDKN2A/B, MLL2, NOTCH1, RB1, TERT* promoter*, TP53*	ICI	PR
25	F	58	Lung, soft tissue	Face skin	High (63)	UV	*CDKN2A, TP53*	EGFR inhibitor	DOD
26	M	75	Lymph node, lung		High (72)	UV	*CDKN2A/B, NOTCH1, RB1, TERT* promoter*, TP53*	ICI	PR

Note: Cases highlighted in italics were identified in the initial review. Surgical resection, radiation, and chemotherapy were omitted from treatment column.

*CR*, Complete remission; *DOD*, dead of disease; *EGFR*, epidermal growth factor receptor; *HHI*, Hedgehog inhibitor; *HPV*, human papillomavirus; *ICI*, immune checkpoint inhibitor; *Muts/Mb*, mutations per megabase; *NP*, not performed; *PR*, partial remission; *SD*, stable disease; *TKI*, tyrosine kinase inhibitor; *TMB*, tumor mutational burden; *UV*, ultraviolet.

Despite clinicopathological challenges, genetic data and clinical courses revealed features unique to BCC. 11 out of 12 BCC cases had corresponding molecular profiles. 10 of 11 cases exhibited high TMB (13 to 106 Muts/Mb) with UV mutational signatures and recurrent *TERT* promoter mutations (10/11) and/or *PTCH1* mutations (9/11). *PTCH1* mutations were exclusively seen in BCC and were highly informative in the context of high TMB and UV mutational signatures. All 12 BCC patients had available clinical follow-up. 11 of 12 BCC patients were treated with ICIs, often in combination with surgery and radiation. 7 patients with BCC also received HhI therapy. Notably, 7 out of 12 (58%) achieved complete remission, while 3 out of 12 (25%) showed partial responses. As a comparison group, metastatic human papillomavirus-associated SCCs with overlapping basaloid cytology and SCCs exhibiting similar high TMB independently or UV-associated SCCs generally exhibited unfavorable outcomes. Of 14 SCC cases, 8 (57%) succumbed to the disease, and none achieved complete remission, despite the use of similar treatment regimens (Fig 1).

Overall, despite the limited scale, our findings highlight therapeutic and prognostic differences between metastatic BCC and SCC. We suggest a low threshold for conducting molecular profiling in cases with atypical clinical presentations and suggestive pathological indications such as prominent basaloid features with the potential for BCC. Presence of high TMB, UV mutational signature, and *PTCH1* mutation should prompt a thorough evaluation for the possibility of metastatic BCC.^3^^,^^4^ If confirmed by clinical and pathological correlation, specific treatment targeting metastatic BCC may be implemented, which can lead to favorable outcomes. More comprehensive studies are needed to investigate the incidence of metastatic BCC in the general population and further explore the clinical impact of this challenging diagnosis.

## Conflicts of interest

None disclosed.
